# Effects of cue focality on the neural mechanisms of prospective memory: A meta-analysis of neuroimaging studies

**DOI:** 10.1038/srep25983

**Published:** 2016-05-17

**Authors:** Giorgia Cona, Patrizia Silvia Bisiacchi, Giuseppe Sartori, Cristina Scarpazza

**Affiliations:** 1 Department of General Psychology, University of Padua, Via Venezia 8, 35131, Padua, Italy; 2 Department of Neuroscience, University of Padua, Via Venezia 8, 35131, Padua, Italy; 3 Cognitive Neuroscience Center, University of Padua, Padua, Italy

## Abstract

Remembering to execute pre-defined intentions at the appropriate time in the future is typically referred to as Prospective Memory (PM). Studies of PM showed that distinct cognitive processes underlie the execution of delayed intentions depending on whether the cue associated with such intentions is focal to ongoing activity processing or not (i.e., cue focality). The present activation likelihood estimation (ALE) meta-analysis revealed several differences in brain activity as a function of focality of the PM cue. The retrieval of intention is supported mainly by left anterior prefrontal cortex (Brodmann Area, BA 10) in nonfocal tasks, and by cerebellum and ventral parietal regions in focal tasks. Furthermore, the precuneus showed increased activation during the maintenance phase of intentions compared to the retrieval phase in nonfocal tasks, whereas the inferior parietal lobule showed increased activation during the retrieval of intention compared to maintenance phase in the focal tasks. Finally, the retrieval of intention relies more on the activity in anterior cingulate cortex for nonfocal tasks, and on posterior cingulate cortex for focal tasks. Such focality-related pattern of activations suggests that prospective remembering is mediated mainly by top-down and stimulus-independent processes in nonfocal tasks, whereas by more automatic, bottom-up, processes in focal tasks.

Prospective Memory (PM) consists in remembering to execute an intention at the appropriate time in the future. In classical event-based PM paradigms, participants are engaged in an ongoing activity and have to simultaneously remember to perform an intention when predefined events or cues occur^1^. Remembering to stop at the grocery store on our way home from work or remembering to give an important message to our colleague are two examples of this kind of PM, and demonstrate the relevance of such function in our everyday life.

Accomplishing a PM task entails multiple phases, which include encoding the intention, maintaining it over time, retrieving and executing it at the right point in time^2^. Furthermore, PM is composed of multiple processes, which are clustered under the terms strategic monitoring and spontaneous retrieval according to the Multiprocess View^1^. Strategic monitoring comprises top-down attentional and memory processes required, respectively, to monitor the environment for PM cues and to maintain the intention active in memory^3^,^4^,^5^. On the other hand, spontaneous retrieval refers to bottom-up attentional and memory processes, which are, for example, automatic capture of attention and activation of intention from mind^1^,^3^,^4^,^6^,^7^. The extent to which PM tasks are supported by strategic monitoring versus spontaneous retrieval processes varies as a function of many different factors^8^,^9^.

Among these factors, one of the most studied is focality of the PM cue, which takes into account the conjoint nature of the PM cue and ongoing activity. A PM task is considered focal when processing of PM cue features is stimulated by processing of the stimuli for the ongoing task (e.g., talking to a friend at a party about theatre is likely to stimulate remembering to pass him the ticket for ‘Romeo and Juliet’ play). Conversely, a PM task is labeled as nonfocal when processing of PM cue features is separated from processing of ongoing stimuli (e.g., talking to a friend at a party about theatre is less likely to stimulate remembering to pass him the ticket for the next baseball game).

According to the Multiprocess View, focal PM cues trigger spontaneous retrieval of the related intentions, whereas nonfocal PM cues encourage the engagement of strategic monitoring processes^1^,^10^,^11^,^12^. Such prediction has been examined and corroborated by several studies^11^,^12^,^13^,^14^,^15^ and focality of the PM cues has been identified as modulating the effect of aging on PM performance^16^,^17^,^18^.

Only recently, have investigations been undertaken in order to explore whether the brain responses to focal and nonfocal PM tasks are characterized by distinct underlying networks. The study of the neural underpinnings of PM has indeed gained an expanding interest in the last decades (e.g.^19^,^20^,^21^,^22^,^23^). Such studies showed the involvement of a widespread number of brain regions to accomplish delayed PM intentions. Within these regions, the anterior prefrontal cortex (aPFC; Brodmann Area, BA 10) was found to play a crucial role in mediating PM tasks^24^,^25^,^26^,^27^,^28^; see^3^ and^19^ for recent reviews). In particular, the lateral parts of the aPFC seem to support stimulus-independent processes, such as maintaining the intention active in mind while being engaged in other ongoing activities, whereas the medial parts of the aPFC seem to mediate stimulus-oriented processes, such as processing of the ongoing stimuli (see also^29^,^30^,^31^).

The frontoparietal networks were also shown essential to PM tasks (e.g.^4^,^32^,^33^). Interestingly, a recent meta-analysis of neuroimaging studies of PM showed a preferential activation of the dorsal and ventral frontoparietal network when considering respectively the phases of PM maintaining and retrieval^3^. More specifically, the dorsal frontoparietal network (i.e., dorsolateral prefrontal cortex (DLPFC), frontal eye fields (FEF), premotor regions, superior parietal lobule and precuneus) was more activated in the maintaining phase. Conversely, the ventral frontoparietal network (i.e., ventrolateral prefrontal regions, supramarginal gyrus and inferior parietal lobule) was more activated during the retrieval phase. Based on these results, the Attention to Delayed Intention (AtoDI) model has been proposed, according to which the dorsal frontoparietal network would support top-down attentional and memory processes required, respectively, to monitor the environment for the PM cue and to maintain the intention active in memory (see also^4^,^6^,^22^,^34^). On the other hand, the ventral frontoparietal network would subserve the bottom-up attention, captured both externally, by the PM cue, and internally, by the representation of the PM cue and the associated intention stored in memory. Together with the ventral frontoparietal network, the insula and the posterior cingulate cortex (PCC) would support processes involved in the retrieval phase, although their functional role is less well-established so far.

To date, only few studies tried to identify the brain mechanisms underlying focal and nonfocal PM tasks, using different techniques and approaches^4^,^13^,^35^. Gordon and colleagues^35^ explored the association between structural parameters and behavioral measures of PM, revealing a positive relationship between the volume of medial temporal regions, such as the hippocampus, and performance on the focal PM task but not on nonfocal task. By investigating the event-related potentials (ERPs), Cona and colleagues^13^ showed that a sustained ERP activity, classically associated with strategic monitoring, was more pronounced in the nonfocal than focal PM task and was particularly expressed over frontal and parietal sites. In contrast, the FN400, an ERP modulation reflecting automatic memory processes, was higher for focal than for nonfocal PM cues, suggesting that the recognition of focal PM cue relies more upon automatic process. Interestingly, McDaniel and colleagues^4^ explored the fMRI correlates of processes implied in focal and nonfocal tasks, providing results that are in line with the AtoDI model^3^. The authors, indeed, found that there are two distinct routes subserving PM. One route is active during the retrieval phase and involves ventral parietal regions, and insular and cingulate cortices. This was interpreted to mediate bottom-up processes. The other route comprises activity mainly in dorsal frontoparietal regions, including DLPFC, FEF and superior parietal lobe, and was interpreted to be involved in top-down monitoring processes. This route was recruited during the maintenance phase, and only for nonfocal PM cues.

A limitation common to all these studies, however, is that the mechanisms required to process the specific features of focal and nonfocal stimuli always differ from each other, thus it is not possible to establish whether the neural differences found in these studies are fully attributable to a variation in the focality dimension or also to the type of process required. For example, in the studies by Gordon *et al*.^35^, and McDaniel *et al*.^4^, participants were required to make category decisions. In these experiments, the focal PM task consisted in remembering to press a key whenever a particular word (e.g., “tortoise”) occurred on the screen, whereas the nonfocal PM task consisted in pressing a key whenever a particular syllable (e.g., ‘tor’) appeared. Hence, detecting the PM cues involved lexical processing for the focal task and sublexical processing for the nonfocal task. In the study by Cona *et al*.^13^ the ongoing activity was a lexical decision task, the focal PM task involved lexical processing whereas the nonfocal task involved semantic processing, as it was required to press a particular key whenever a word belonging to a given target category occurred on the screen (e.g., “daisy” for the category “flower”).

A recent model - Dual Pathways model - proposed by McDaniel and collaborators^36^ made several predictions about the possible neural regions that would differentially support focal and nonfocal PM tasks on the basis of the multiprocess framework. According to the authors, nonfocal PM tasks would involve both strategic monitoring, which would be mediated by activation of DLPFC, VLPFC, insula, anterior cingulate cortex (ACC), lateral BA 10, and precuneus; and intentional retrieval, which would be supported by inferior parietal lobe (BA 40), insula, lateral BA 10 and ACC. By contrast, focal tasks would stimulate spontaneous retrieval, thus they would involve activation of ventral frontoparietal regions, BA 9, and medial temporal regions. Although insightful, such considerations were however driven by speculative inferences derived from the findings in the single studies. In such a way, the proposed model could not overcome the specificities of the stimuli and tasks employed in each study.

The present study used a meta-analytic approach to delineate brain regions that are differentially activated in focal and nonfocal PM tasks, looking beyond the idiosyncrasies of the individual experiment. In this sense, the meta-analysis allowed us to identify the ‘core’ neural mechanisms that are specific for focal and nonfocal PM tasks, regardless of the specific nature of stimuli and tasks adopted by the single experiment.

As the AtoDI and Dual Pathways models share many similarities, several hypotheses can be made following such models^3^,^36^. We indeed predicted that the dorsal frontoparietal network, being associated with top-down processes as strategic monitoring, would be active mainly in the maintenance phase and especially for the nonfocal PM tasks. Conversely, the ventral frontoparietal network, subserving bottom-up retrieval, would be active mainly in the retrieval phase and especially for the focal PM tasks. Nevertheless, in contrast to the Dual Pathways model, we hypothesized that the inferior parietal lobe (BA 40), mediating bottom-up attentional and memory processes, would be activated also in focal tasks.

Retrieval in the nonfocal tasks would be instead more intentional, thus it would be supported by different structures, such as the ACC and lateral regions of the aPFC. Indeed, if the lateral aPFC mediates stimulus-independent processes, then a greater involvement of such region would be expected for nonfocal tasks, wherein processing of the PM cue is independent from processing of ongoing external stimuli, compared to focal tasks, wherein there is a closer overlap between processing of the ongoing and PM stimuli.

## Results

### Activations in nonfocal PM tasks

**Figure 1:**
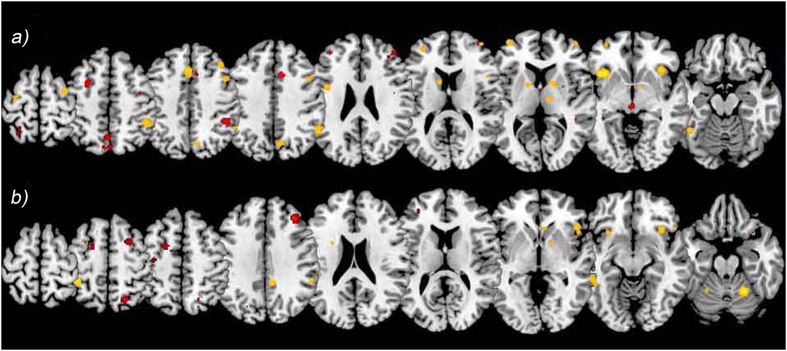
Foci of activation in the maintenance phase (in red) and retrieval phase (in yellow), for both nonfocal tasks and focal tasks. (**a**) Nonfocal tasks, z = {271, 241, 226, 218, 192, 169, 153, 130, 108}); (**b**) Focal tasks, z = {270, 255, 246, 209, 184, 166, 136, 119, 99}.

The meta-analysis on maintenance phase for nonfocal tasks included 95 foci from 11 experiments, and a total of 191 subjects. In the maintenance phase of nonfocal PM paradigms activations were located mainly in dorsal regions of frontal lobe, in particular in the middle and superior frontal gyrus (BA 6 and 9 respectively), and extensively in the posterior parietal cortex regions, comprising inferior and superior parietal lobules (BA 40, BA 7) and precuneus (BA 7). The anterior cingulate cortex (BA 32) and subcortical regions, as the red nucleus, were activated as well. Results are reported in Table 1 and Fig. 1.

The meta-analysis on retrieval phase for nonfocal tasks included 193 foci from 14 experiments, and a total of 274 subjects. Retrieving an intention when a nonfocal cue occurs has been found to be mainly associated with activations in the bilateral insular regions (BA 13), in the lateral aPFC regions (BA 10, BA 9), in right ventral frontal regions (BA 45) and in premotor cortex (BA 6). Activations in parietal cortex were mainly located in the inferior parietal lobule (BA 40) and precuneus (BA 7). Clusters of activations were also found in the ACC (BA 32) and in the fusiform gyrus (BA 37). Subcortical regions were also activated, in particular the thalamus and caudate (Table 1 and Fig. 1).

### Nonfocal PM tasks: Direct comparison between the maintenance and retrieval phase

**Figure 2:**
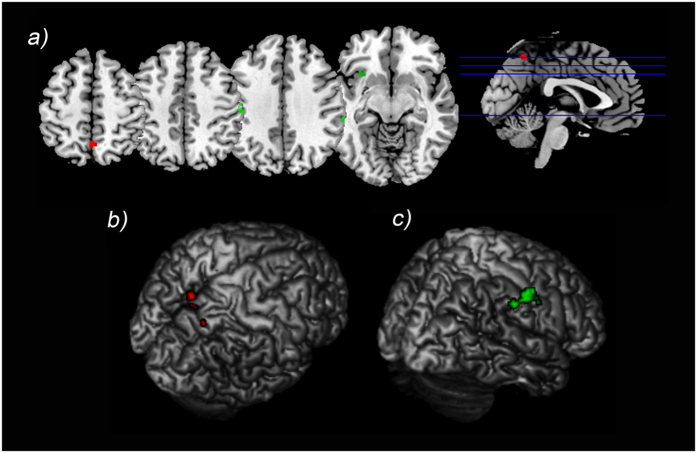
Foci of activation from the direct comparison between the maintenance and retrieval phase for nonfocal and focal tasks. (**a**) Foci of activation from the direct comparison between the maintenance and retrieval phase presented in the axial plane (z = {126, 117, 108, 63}). The foci of activation for the contrast maintenance >retrieval phase when a nonfocal cue is provided are reported in red. The opposite comparison (retrieving >maintenance) when a nonfocal cue is provided did not reveal any significant result. The foci of activation for the contrast retrieval >maintenance phase when a focal cue is provided are reported in green. The opposite comparison (maintenance >retrieval) when a focal cue is provided did not reveal significant results. (**b**) Maintenance >Retrieval with nonfocal cues results were projected onto a surface rendering in order to visually appreciate the activation in the precuneus (BA 7); (**c**) Retrieval >maintenance with focal cues results were projected onto a surface rendering in order to visually appreciate the activation in inferior parietal lobule (BA 40).

To further explore the possible dissociation between the maintenance and retrieval phases in nonfocal tasks, a direct comparison between the previous reported maps of activation was performed. The precuneus (BA 7) showed increased activation during the maintenance phase compared to the retrieval phase. By contrast, no significant results emerged from the opposite comparison (retrieval >maintenance). Results are shown in Table 2 and Fig. 2.

### Activations in focal PM tasks

The meta-analysis on maintenance phase for focal tasks included 53 foci from 6 experiments, and a total of 110 subjects. This analysis revealed consistent activations in the frontal regions, including the FEF, premotor and motor areas (respectively BA 8, BA 6, BA 4) and activations in both dorsal and ventral parietal regions (BA 7, BA 40). Results are reported in Table 1 and Fig. 1.

The meta-analysis on retrieval phase for focal tasks included 122 foci from 7 experiments, and a total of 163 subjects. Such analysis showed activations located mainly in the cerebellum, and in the frontal and parietal lobe. Among frontal regions, consistent activations have been found in inferior frontal regions (BA 45, BA 47, BA 9) and in precentral gyrus (BA 4, 6). Smaller activations were also found in the FEF (BA 8). Among parietal regions, the inferior parietal lobule and supramarginal gyrus (BA 40), and post-central gyrus (BA 2) were found consistently activated. Additional activations have been found in insula (BA 13), in PCC (BA 31) and in subcortical structures, as caudate and subthalamic nucleus (Table 1 and Fig. 1).

### Focal PM tasks: Direct comparison between maintenance and retrieval phase

To further explore the possible dissociation between the maintenance and retrieval phase of intention for focal PM tasks, a direct comparison between the previous reported maps of activation was performed. The inferior parietal lobule (BA 40) showed increased activation during the retrieval of intention compared to maintenance, as well as insula and post-central gyrus (BA 2). By contrast, no significant results emerged from the opposite comparison (maintenance >retrieval). Results are shown in Table 2 and Fig. 2.

### Direct comparison between nonfocal and focal PM tasks

**Figure 3:**
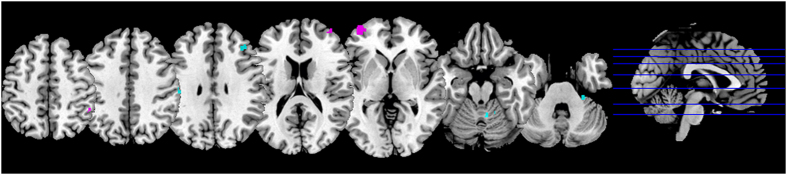
Foci of activation of the direct comparison between nonfocal and focal PM tasks (z = {237, 223, 199, 171, 140, 97, 76}). The foci of activation referring to the contrast nonfocal tasks >focal tasks are reported in pink and revealed that nonfocal tasks are associated with increased activity in lateral aPFC regions (BA 10). The foci of activation referring to the contrast focal tasks >nonfocal tasks are reported in light blue and showed that focal tasks are associated with increased activity in the anterior cerebellum, in the ventral parietal regions (BA 40) and in the BA 9.

In order to better highlight brain activations that are expressed differentially in nonfocal and focal tasks, we first computed an analysis of the activation foci pooling together the maintenance and retrieval phases; then we analyzed such activations separately for each phase. Interestingly, as compared with focal tasks, nonfocal tasks were associated with an extensive cluster of increased activity in the lateral aPFC and DLPFC regions, over the left hemisphere (Table 3, Fig. 3). Notably, the analysis of activity separately for each PM phase revealed that lateral aPFC regions only were more active in nonfocal than focal phase (MNI coordinates: −30, 53, 0; BA = 10; *p* = 0.024). No significant focality-related differences were found in the maintenance phase.

On the other hand, increased activations in focal PM tasks than in nonfocal PM tasks were shown in the anterior lobe of the cerebellum, in the ventral parietal regions (BA 40) and the middle frontal gyrus (BA 9). Results are shown in Table 3 and in Fig. 3. The analysis of activity separately for each PM phase revealed differences only in the retrieval phase, with focal tasks being associated with increased activations in PCC (8, −43, 37; BA 31; *p* = 0.023), in the inferior parietal lobule (65, −30, 36; BA 40; *p* = 0.026), in supramarginal gyrus (63, −24, 40; BA 40; *p* = 0.029), in the cerebellum (4, −66, −18; *p* = 0.028), and in motor regions (64, −14, 36; BA 4; *p* = 0.025). No significant focality-related differences were observed in the maintenance phase.

## Discussion

The goal of the present study was to identify brain structures that are consistently activated to accomplish focal and nonfocal PM tasks, regardless of the specific nature of the stimuli utilized for the ongoing and PM tasks.

The first finding, which corroborates previous results^6^,^7^,^8^,^9^,^10^,^11^,^12^,^13^,^14^,^15^,^16^,^17^,^18^,^19^,^20^,^21^,^22^,^23^,^24^,^25^,^26^,^27^,^28^,^29^,^30^,^31^,^32^,^33^,^34^,^35^,^36^,^37^, concerns the differential involvement of dorsal and ventral frontoparietal networks during the maintenance and retrieval PM phases. In general, for both focal and nonfocal tasks, the dorsal frontoparietal network, comprising regions as DLPFC, FEF, premotor regions, precuneus and superior parietal lobule (BA 7), was involved to a greater degree in the maintenance phase, whereas the ventral frontoparietal network, including ventrolateral frontal regions (BA 45, BA 47) and the inferior parietal lobule (BA 40) was involved to a greater degree in the retrieval phase. According to the AtoDI model^3^, the dorsal frontoparietal network would be more active in the maintenance phase as it mediates the allocation of top-down attention toward both the external ongoing stimuli for checking the PM cue occurrence and toward the intention so as to keep it actively in mind. On the other hand, the ventral frontoparietal network is mainly required during the retrieval phase as it subserves the bottom-up allocation of attention, captured externally by the PM cue and internally by the representation of the associated intention. It is important noting that the results of our previous work^3^ and the current one are based on the same literature, with the only difference that the current work included also the most recent papers, which were not included in the former analysis.

The second finding, more relevant for the purpose of the current study, was the differential recruitment of parietal regions as a function of the focality of the PM task. Indeed, for the nonfocal PM tasks, increased activity in the precuneus (BA 7) was observed in the maintenance phase compared to the retrieval phase, whereas no significant activity increase was found in the retrieval phase compared to the maintenance phase. Conversely, for the focal PM tasks, increased activity of the inferior parietal lobule (BA 40) was shown during the retrieval phase compared to maintenance phase, whereas no activity increase was found considering the opposite contrast. Notably, the inferior parietal lobule was found to be more active at retrieval for focal tasks than for nonfocal tasks. This result is in line with the AtoDI model, which predicted that the recruitment of dorsal and ventral parietal regions can be modulated by the features of the PM cues, as focality (Paragraph 4.5^3^). It indeed suggests that, when facing up to nonfocal PM tasks, individuals tend to rely mainly upon top-down processes, such as maintaining and refreshing the intention in memory, which seem to be mediated by the precuneus^3^,^4^,^38^; whereas they mainly rely upon bottom-up attention and memory processes, mediated by the inferior parietal lobule (BA 40) and deployed during the retrieval phase, when accomplishing focal PM tasks (see also^4^,^6^,^28^ for similar interpretations). Yet, this result arguments against the predictions made by Dual Pathways model^36^, according to which BA 40 would be involved in the intentional retrieval, and it would be active only in nonfocal tasks, not in focal tasks.

Together with differences, it is important to underline that there were also similar activation patterns for focal and nonfocal PM. Indeed, the dorsal frontoparietal network was active during the maintenance phase also in focal PM tasks. The involvement of such network could be due to the fact that, although not needed, strategic monitoring processes were recruited also to accomplish focal PM tasks, as demonstrated by previous studies that found monitoring costs even in such tasks^4^,^13^. This activation might be due to the fact that the tasks utilized in the fMRI studies typically employed relatively frequent PM cues. This would emphasize the allocation of strategic monitoring resources since individuals tend to engage monitoring when expecting the occurrence of the PM cue^39^,^40^,^41^.

The third relevant finding was that a significant increased activity in nonfocal tasks compared to focal PM tasks was found in the lateral aPFC regions (BA 10), especially of the left hemisphere. In line with the Gateway hypothesis^19^,^42^, the aPFC regions would mediate stimulus-independent processing, biasing the processes towards internal representations. These processes would be particularly required in nonfocal tasks, where processing of PM cue features is (at least partially) independent from processing of ongoing stimuli. Furthermore, as shown by the analysis of functional connectivity performed by McDaniel and colleagues^4^, the aPFC has stronger connectivity with the precuneus in the nonfocal PM task and this interaction was interpreted to support retrieval mode.

By contrast, as compared with nonfocal PM tasks, focal tasks were associated with greater activation in a more posterior region (i.e., BA 9). Such region has been previously found associated with involuntary episodic retrieval^43^, thus it has been suggested that it might play a similar role in PM tasks as well^36^. Moreover, enhanced activity for focal tasks (than nonfocal tasks) was found in the right cerebellum. Although many studies of PM have shown an involvement of cerebellum regions^44^,^45^, the role of such regions is still a largely neglected question, thus the explanations provided below are, at this point, essentially speculative, and based on the reverse inference. The cerebellum has been typically associated with motor processes, as motor coordination and learning^46^,^47^. Previous studies have also highlighted the critical role of cerebellum in motor automaticity^48^,^49^. More specifically, it might be meaningful to take into account the suggestion provided by Swinnen and colleagues^50^, according to which the cerebellar route mediates implicit fast processing of motor intention whereas the parietal route mediates explicit monitoring of intention and motor scheme at higher levels (see also^51^). This interpretation could be applied in the PM context as well, especially given that such involvement was observed in the retrieval phase. In this sense, the focal PM tasks would stimulate the recruitment of cerebellar regions, which would support automatic activation of the motor plan of the PM intention. Recently, several studies provided functional neuroimaging evidence for cerebellar involvement in non-motor functions, such as cognition and emotion^52^,^53^. Hence, it is possible to hypothesize that the cerebellum is relevant for supporting cognitive mechanisms in the focal PM tasks. Nevertheless, identifying the precise role of the cerebellar regions in cognition – and more specifically in prospective remembering – remains a challenge for future investigations.

Notably, differential involvement of anterior *versus* posterior cingulate cortices as a function of PM cue focality was also observed. More specifically, nonfocal tasks were associated with activation of ACC, in both the maintenance and the retrieval phase. Based on the AtoDI model, the ACC – being related to conflict monitoring and cognitive control – would monitor and signal the potential competition between ongoing and PM task rules, and would send such signal to lateral aPFC regions, which support biasing of attention between ongoing and PM task rules. As such, and in line with the predictions of McDaniel *et al*.^36^, the ACC contributes to strategic monitoring, which is critically involved in nonfocal tasks. By contrast, focal tasks were associated with activation in PCC, in the retrieval phase. According the AtoDI model, the PCC would mediate bottom-up processing, cooperating with inferior parietal regions in shifting attention from the external PM cue to the internal to-be-retrieved intention^3^. Furthermore, consistent with the present finding, several studies on incidental memory retrieval showed that the PCC is activated automatically and independently of the intention to retrieve a given episode^54^,^55^. This interpretation well fit with the finding of a selective transient activation of this region in the retrieval phase of focal tasks.

Other two brain structures appear to be differentially involved in focal and nonfocal PM tasks during the retrieval phase: These are the right middle temporal (BA 21) and insular (BA 13) regions (Table 1). The right middle temporal regions were recruited to a greater degree in the retrieval phase of focal PM tasks. Such activation has been interpreted to reflect processes related to PM cue detection^4^,^23^. In particular, McDaniel and colleagues^4^ suggested that middle temporal regions serve the suspension of processing of the ongoing stimuli and shifting attentional focus toward the intention-related significance of the PM cue. We did not find instead consistent activations within medial temporal (MTL) regions. The contribution of such areas to PM is however still controversial, with past available studies producing mixed results (e.g.^3^,^4^,^5^,^23^,^44^).

Conversely, bilateral insular regions were recruited to a greater degree in nonfocal PM tasks, and selectively in the retrieval phase. Such result indicates that insula does not mediate sustained processes, as previously proposed^36^, but is transiently activated only when the PM cue occurs. The insula belongs to the “Salience Network”, which is considered to enable the detection of relevant external or internal stimuli to guide behavior and thoughts^56^. Based on this, in our previous meta-analysis, we proposed that the insular regions may play a role in detecting the presence of the PM cues in the environment and in informing about these relevant stimuli the connected areas, which would bias processing of stimuli accordingly^3^. Although insular regions are involved in both kinds of tasks, the extent to which they are recruited seems to vary as a function of cue focality, being more extensively activated in tasks with nonfocal cues. As previously proposed, the insula might be essential for making stimuli as ‘salient’, and this would require further access to working memory and attentional resources^57^,^58^. A recent study also showed activation of the bilateral dorsal anterior insula in a PM task that comprised endogenous cues^57^. On the basis of these findings, the greater involvement of insular regions for nonfocal tasks would reflect the greater amount of attentional and memory resources required to make nonfocal cues as salient, and thus, more likely to be detectable. Notably, a number of studies postulated that the anterior insula is devoted to very general processes of top–down control and attention (e.g.^59^,^60^). Together with the ACC, indeed, the anterior insula forms the core of the network for cognitive control, which would be required especially for nonfocal tasks^59^. An alternative view suggested that the anterior insula would provide interoceptive signals that make it possible to evaluate the outcomes and the consequences of intentional actions^61^. This interpretation is consistent with the pattern of focality-related ERP differences found in the study by Cona *et al*.^13^, which indicated that nonfocal PM tasks involve a greater recruitment of strategic resources not only to monitor for the occurrence of the PM cue, but also to monitor the outcome of the retrieved intention. Future studies are however needed to clarify the functional contribution of the insula to PM.

Since the study of the neural correlates of PM is a recent field of investigation, the neuroimaging studies that sought to address this issue are relatively few (but see^62^,^63^, for a similar number of studies). Furthermore, so far only one study has directly explored the differences in brain activity between focal and nonfocal tasks^4^. This led to two main limitations in the present study. First, the direct contrasts (e.g., between focal and nonfocal tasks for the maintenance and retrieval phases) suffered for lower statistical power, thus the results obtained from such analyses need further investigation and should be considered with caution. Second, since the role of some brain regions has not been clearly investigated and identified by previous studies, some of our interpretations (e.g., the role of cerebellum) were driven by reverse inference, and therefore should be considered speculative. Despite these limitations, this meta-analysis of functional neuroimaging studies has the advantage to statistically test the predictions made by the AtoDI^3^ and the Dual Pathways models^36^, giving supports to some of them and reviewing others, and can drive and assist the research community to highlight differences and commonalities in brain activation underpinning focal and nonfocal PM tasks.

To sum up, we have found that frontoparietal networks play a key role in both focal and nonfocal PM tasks but the degree to which dorsal and ventral frontoparietal regions are recruited varies as a function of both the phase and focality of PM task. Furthermore, we found differences in brain activation as a function of PM cue focality especially during the retrieval phase: Focal tasks evoke greater activation in the cerebellum, in ventral parietal regions and within the BA 9, whereas nonfocal tasks rely more upon activation in the lateral parts of the BA 10 and in the insula. Finally, differential activations in the cingulate cortices depending on PM cue focality were observed, with the ACC being involved in nonfocal tasks and the PCC being involved in focal tasks. Focality-related differences were observed mainly in the retrieval phase, supporting the hypothesis that retrieval is more spontaneous for focal tasks and more controlled and intentional for nonfocal tasks^36^. They also lead to important modifications to existing models, re-defining some interpretations of the neuroimaging results^3^,^36^.

## Material and Methods

### Study Selection

Several online electronic databases (e.g., Psycinfo, Medline, PubMed) were searched until April 2015 in order to identify studies that are suitable to be included in the current meta-analysis. Combinations of relevant terms (e.g., “prospective memory”, “delayed intention”, “future intention”, “neuroimaging”, “fMRI”, “functional magnetic resonance imaging”, “focal”, “nonfocal”) were used as search filter. Furthermore, additional studies were found using the “related articles” function of the PubMed database and tracing the references from review articles and the identified papers. The following inclusion criteria were used to select articles:

1. Included articles should use PET or fMRI, both blocked and event-related, methodology;
2. Included articles should present the results of the whole brain analysis, while articles presenting only ROI analysis were excluded;
3. Included articles should adopt event-based PM tasks, while articles presenting only time-based PM studies were excluded since they are a particular type of PM, mediated by distinct processes;
4. Included articles should describe a clear contrast representing locations of greater activation for PM conditions as compared with control conditions. To explore activity in the maintenance phase, contrasts were usually performed between ‘baseline blocks’, in which the ongoing task was performed alone (e.g., same-different judgments, N-back tasks) and ‘PM blocks’, in which ongoing task and PM task were concurrently executed. To investigate activity in the retrieval phase, ongoing trials during PM blocks were usually compared with PM trials.
5. Included articles should report activation’s peaks in a standardized coordinate space (e.g., Talairach and Tournoux, 1988, or MNI). Tailarach coordinates were then reported into MNI space before performing the meta-analysis using a linear transformation^64^,^65^.
6. Included articles should be peer-reviewed articles reporting novel data, while replication studies have been excluded.
7. Included studies should have a sample size of at least 5 participants.

Based on these criteria, 22 studies were found to be eligible for inclusion into the meta-analysis (cf. Table 4). Together, these studies reported 463 activation foci obtained from 38 individual experiments (with “study” referring to a paper, and “experiment” referring to an individual contrast reported in this paper) representing regions of significantly greater activation in the PM task as compared with the control task.

### Classification of the studies

To classify the studies as using focal or nonfocal PM tasks, we evaluated the extent to which processing of the ongoing stimuli and processing of the PM cues overlap in each experiment, according to the descriptions of Einstein and McDaniel^1^,^11^ (i.e., focal cues are PM cues that overlap with the information relevant to performing the ongoing activity, nonfocal cues are PM cues that are present in the environment but not part of the information being processed for the ongoing activity). It must be said that a clear-cut distinction of focal and nonfocal tasks is not possible, as the dimension of PM cue focality cannot be conceptualized as a dichotomy but rather along a continuum. Nevertheless, our classification of the studies matched that proposed by McDaniel *et al*.^36^, for all but two studies: the studies by Rea *et al*.^66^ and by Benoit *et al*.^67^. More specifically, in the study by Rea *et al*.^66^, participants were required to respond to specific face stimuli (PM task) while being engaged in a same/different gender face judgment task (ongoing task). We considered this PM task as focal since the PM task/ongoing task combination, although including pictures instead of words, represents a close parallel to the most common combination used for exploring focal PM tasks, in which participants were required to respond to specific words (PM task) while being engaged in category decision tasks (ongoing task)^4^,^10^,^35^. The classification of the study by Benoit *et al*.^67^ was more complicated. We classified such study as nonfocal following the task description reported by the authors themselves, who stated that the PM cues were chosen so that the PM task was not so easily incorporated in the ongoing task. Furthermore, in line with the encoding specificity principle, McDaniel and collaborators proposed that focal processing occurs when ‘there is strong overlap between how a cue was processed at encoding and at retrieval’^36^, which was rarely the case in this experiment. Indeed, first, the PM cue was often externally presented in the encoding phase, but internally processed in the retrieval phase; and second, in the ‘alphabet’ task, the PM cue was encoded as a word, but presented as a letter in the retrieval phase, while participants were engaged in classifying letters for straight or curved lines in the ongoing task.

Based on such classification, 288 foci from 25 experiments using nonfocal tasks were obtained, while 175 foci from 13 experiments using focal tasks were obtained.

### ALE consistency analysis

We used the optimized version of ALE algorithm (ALE-S^68^) for coordinate-based meta-analysis of neuroimaging results to generate Activation Likelihood Estimation maps based on the activation peak. This approach allows researchers to identify areas with a convergence of reported coordinates across experiments that is higher than expected from a random spatial association. The exact procedure was explained in detail elsewhere^68^,^69^,^70^. Briefly, this algorithm considers activated foci of brain regions as three dimensional Gaussian probability distributions centered at the given coordinates instead of points^69^,^71^. Importantly, the sample size of each study was considered in the algorithm with the aim to incorporate the size of the probability distributions. Indeed, since smaller effect size can be potentially associated with studies having larger sample size^68^,^69^, it is important to take into account this information. Moreover, the random-effect was used by the algorithm rather than the fixed-effect inference. This was implemented by testing the above chance clustering between experiments/contrasts rather than the above-chance clustering between foci. Inference is then sought regarding regions where the likelihood of activation being reported in a particular set of experiments is higher than expected by chance, i.e., where there is a non-random convergence. Importantly, this inference is performed against an appropriate null-hypothesis reflecting random spatial association.

We used the optimized version of ALE algorithm (ALE-S^68^) since it prevents multiple experiments performed by the same group of participants from cumulatively influencing ALE values. Indeed, it is frequent that one single study includes multiple distinct experiments, which are considered as independent experiments by GingerALE, but are, instead, not independent as they share the same participants.

Consistency analyses have been performed on different PM phases (maintenance and retrieval) and for different task focality (nonfocal and focal). A total of four ALE consistency analyses were created, in order to highlight the brain regions that are consistently activated during the maintaining phase in nonfocal PM tasks; during the retrieval phase of nonfocal PM tasks; during the maintaining phase in focal PM tasks and during the retrieval phase of focal PM tasks. The p-value of the ALE score was given by the proportion of equal or higher values obtained under the null distribution, which reflects a random spatial association among foci. For consistency with the previous meta-analysis on the same topic^3^, the resulting non-parametric p values were then assessed at a false discovery rate (FDR) corrected threshold of p < 0.05 on cluster level and transformed into Z scores for display^71^. Moreover, an extent threshold of k > 50 voxels was applied to the results. GingerALE 2.3 software (http://www.brainmap.org/ale/) was used for the analysis.

### ALE discriminability analyses

Discriminability analyses were conducted in order to directly compare two individual ALE maps. This analysis enables the identification of statistical differences between two ALE results. In particular, the discriminability analysis has been used to test the existence of possible statistical significant differences between maintenance and retrieval of intention (i.e., maintenance > retrieval and retrieval > maintenance) separately for focal and nonfocal tasks. In addition, the same analysis was used to understand if statistical differences exist in brain activations depending on the focality of the cue, first, regardless of the PM processes (focal >nonfocal and nonfocal >focal, pooling the activation for maintenance and retrieval together) and then, separately for each phase.

Statistical differences between two ALE maps were tested by first performing separate ALE meta-analyses for the two information that need to be directly compared. The experiments contributing to either analysis were then pooled and randomly divided into two groups of the same size as the sets of contrasted experiments^72^. Voxelwise ALE scores of these two randomly assembled groups were subtracted from each other and recorded. Repeating this process 10,000 times yielded an empirical null distribution of ALE-score differences between the two conditions. Based on this permutation procedure, the map of true differences was then thresholded at a posterior probability of p > 0.99 for a true difference between the two samples (p < 0.01 uncorrected). In addition, a cluster extent threshold of k > 50 was applied to eliminate minor, presumably incidental, findings.

## Additional Information

**How to cite this article**: Cona, G. *et al*. Effects of cue focality on the neural mechanisms of prospective memory: A meta-analysis of neuroimaging studies. *Sci. Rep.* **6**, 25983; doi: 10.1038/srep25983 (2016).

## Figures and Tables

**Table 1:** Foci of activation separately for the distinct PM phases (maintaining and retrieval of intention) and for the focality of the cue (focal or nonfocal).

**Cluster Size (mm3)**	**Brain regions**	**Brodmann Areas**	**MNI coordinates**			**ALE p-value**
			**x**	**y**	**z**	
*Nonfocal PM tasks: Maintenance phase*						
688	Middle frontal gyrus	6	−28	6	50	0.011
	Middle frontal gyrus	6	−32	0	56	0.011
624	Supramarginal gyrus	40	46	−42	42	0.012
600	Precuneus	7	−2	−62	48	0.011
448	Inferior parietal lobule	40	−50	−36	40	0.009
	Inferior parietal lobule	40	−46	−36	44	0.009
448	Precuneus	7	−6	−74	52	0.010
	Precuneus	7	0	−74	52	0.009
384	Anterior Cingulate gyrus	32	10	16	38	0.012
312	Red nucleus	−	0	−22	−4	0.010
136	Superior frontal gyrus	9	42	44	26	0.010
88	Precentral gyrus	9	−54	10	32	0.009
80	Superior parietal lobule	7	−26	−56	66	0.008
*Nonfocal PM tasks: Retrieval phase*						
1768	Insula	13	−38	18	−6	0.023
1280	Middle frontal gyrus	10	−38	56	2	0.019
976	Medial frontal gyrus	6	−2	22	44	0.019
736	Insula	13	36	24	−4	0.019
640	Supramarginal gyrus	40	56	−52	38	0.015
608	Inferior parietal lobule	40	46	−42	52	0.015
592	Middle frontal gyrus	10	−34	50	18	0.015
536	Thalamus	—	−14	4	4	0.011
	Caudate	—	−12	8	14	0.010
424	Insula	13	50	14	10	0.012
	Inferior frontal gyrus	45	48	24	10	0.010
408	Middle frontal gyrus	8	38	30	44	0.016
392	Caudate	—	14	6	8	0.012
376	Precuneus	7	8	−68	40	0.012
368	Superior frontal gyrus	10	42	56	10	0.011
368	Precentral gyrus	6	−40	2	28	0.013
352	Precentral gyrus	9	44	12	40	0.014
336	Insula	13	56	−18	20	0.014
336	Middle frontal gyrus	6	30	−4	64	0.014
328	Fusiform gyrus	37	−42	−52	−16	0.013
320	Superior frontal gyrus	10	28	54	−12	0.013
312	Supramarginal gyrus	40	−58	−46	38	0.013
224	Anterior Cingulate gyrus	32	−6	30	30	0.011
184	Thalamus	—	10	−14	6	0.012
144	Precentral gyrus	6	−30	−10	64	0.009
128	Inferior parietal lobule	40	−42	−40	60	0.010
120	Superior frontal gyrus	10	−20	48	20	0.009
	Medial frontal gyrus	9	−18	44	20	0.008
104	Inferior parietal lobule	40	−62	−22	28	0.010
72	Middle temporal gyrus	22	−60	−52	6	0.010
*Focal PM tasks: Maintenance phase*						
936	Middle frontal gyrus	8	36	36	34	0.014
528	Middle frontal gyrus	6	−20	2	54	0.013
520	Superior parietal lobule	7	20	−62	56	0.013
448	Middle frontal gyrus	6	26	6	58	0.011
288	Precentral gyrus	4	−36	−18	54	0.008
280	Occipital lobe	19	−22	−74	40	0.008
160	Inferior parietal lobule	40	−44	−34	52	0.008
136	Inferior parietal lobule	40	34	−46	42	0.008
128	Supramarginal gyrus	40	42	−44	38	0.008
104	Anterior Cerebellum Lobe	—	30	−54	−30	0.008
96	Middle frontal gyrus	10	−34	44	14	0.008
*Focal PM tasks: Retrieval phase*						
904	Anterior Cerebellum Lobe	—	22	−54	−20	0.019
880	Inferior parietal lobule	40	66	−34	38	0.013
	Postcentral gyrus	2	60	−20	44	0.011
	Precentral gyrus	4	62	−14	38	0.009
680	Inferior frontal gyrus	45	46	24	−2	0.012
560	Inferior parietal lobule	40	−38	−42	58	0.015
544	Inferior frontal gyrus	47	34	22	−10	0.012
520	Middle temporal gyrus	21	64	−38	−4	0.011
	Middle temporal gyrus	21	68	−44	−4	0.009
488	Precentral gyrus	6	−58	6	38	0.012
	Inferior frontal gyrus	9	−56	10	28	0.010
448	Posterior Cingulate gyrus	31	8	−42	36	0.013
416	Supramarginal gyrus	40	−46	−38	42	0.013
272	Middle frontal gyrus	8	−40	28	44	0.011
240	Anterior Cerebellum Lobe	—	28	−30	−34	0.009
136	Insula	13	−30	18	−10	0.010
136	Inferior parietal lobule	40	56	−40	34	0.010
96	Thalamus	—	−6	−12	−6	0.009
80	Caudate	—	6	24	−2	0.008
80	Inferior occipital gyrus	19	−36	−76	0	0.008

**Table 2:** Foci of activation of the direct comparison between the maintenance and retrieval phases in nonfocal and focal PM tasks.

**Cluster Size (mm3)**	**Brain regions**	**Brodmann Areas**	**MNI coordinates**			**ALE p-value**
			**x**	**y**	**z**	
*Nonfocal tasks: Maintenance* >*Retrieval*						
232	Precuneus	7	−1	−57	52	0.031
*Nonfocal tasks: Retrieval* >*Maintenance*						
	no significant activations detected					
*Focal tasks: Maintenance* >*Retrieval*						
	no significant activations detected					
*Focal tasks: Retrieval* >*Maintenance*						
264	Postcentral gyrus	2	60	−20	44	0.038
	Inferior Parietal Lobule	40	62	−29	39	0.025
128	Anterior Insula	13	−30	17	−10	0.025

**Table 3:** Foci of activation of the direct comparison between nonfocal and focal PM tasks.

**Cluster Size (mm3)**	**Brain regions**	**Brodmann Areas**	**MNI coordinates**			**ALE p-value**
			**x**	**y**	**z**	
*Nonfocal tasks* >*Focal tasks*						
1056	Middle frontal gyrus	10	−32	54	−4	0.029
	Middle frontal gyrus	10	−35	50	−2	0.027
	Middle frontal gyrus	46	−40	50	−2	0.027
	Middle frontal gyrus	10	−38	52	1	0.026
	Middle frontal gyrus	10	−40	56	1	0.025
	Middle frontal gyrus	10	−37	60	5	0.027
*Focal tasks* >*Nonfocal tasks*						
928	Middle frontal gyrus	9	39	35	34	0.025
152	Anterior Cerebellum lobe	—	28	−32	−32	0.027
	Anterior Cerebellum lobe	—	28	−28	−33	0.024
144	Supramarginal gyrus	40	63	−24	40	0.025
	Supramarginal gyrus	40	62	−24	44	0.025
120	Anterior Cerebellum lobe	—	8	−54	−20	0.029

**Table 4:** Studies included in the meta-analysis. NA = not available.

**First Author**	**Year**	**Number of Subjects**	**Number of PM trials (and %)**	**Contrast**	**Foci (n)**	**PM phase**		**Cue Focality**	
						**M**	**R**	**NF**	**F**
Okuda^27^	1998	6	2–3 (5%)	Maintenance	7	x			x
Burgess^73^	2001	8	96 (20%)	Maintenance	10	x		x	
				Retrieval	1		x	x	
Burgess^74^	2003	9	NA (20%)	Maintenance	1	x		x	
Den Ouden^24^	2005	11	NA (23%)	Maintenance	3	x		x	
Simons^75^	2006	16	32 (NA)	Maintenance	2	x		x	
				Retrieval (stimulus with high identification demand)	16		x	x	
				Retrieval (stimulus with high retrieval demand)	29		x	x	
Okuda^45^	2007 EX1	10	5 (12.5%)	Maintenance (event-related)	10	x			x
Gilbert^25^	2009	16	NA (5.6%)	Maintenance	13	x		x	
				Retrieval (self initiated cue)	14		x	x	
				Retrieval (external cue)	6		x	x	
Kalpouzos^33^	2010	14	22 (NA)	Maintenance	24	x			x
				Retrieval	26		x		x
				Retrieval	16		x		x
Benoit^67^	2011	16	NA (11%)	Maintenance	7	x		x	
Gilbert^44^	2011	32	60 (8%)	Maintenance	5	x			x
				Retrieval	5		x		x
Rea^66^	2011	13	52 (3%)	Retrieval (neutral stimulus)	25		x		x
				Retrieval (emotional stimulus)	33		x		x
Rusted^28^	2011	8	32 (7.6%)	Retrieval	7		x	x	
Hashimoto^76^	2011	16	15 (5.8%)	Maintenance	17	x		x	
				Retrieval	22		x	x	
				Retrieval	28		x	x	
Okuda^77^	2011	16	120–160 per task (≈ 6.6%)	Maintenance	9	x		x	
				Retrieval in expanding condition	4		x	x	
				Retrieval in contracting condition	3		x	x	
Gilbert^21^	2012	32	60 (8%)	Maintenance	2	x			x
				Retrieval	7		x		x
McDaniel^4^	2013	45	20 (4%)	Maintenance	7	x		x	
				Retrieval	10		x		x
				Retrieval	10		x	x	
Gonneaud^22^	2013	20	15 (12.5%)	Maintenance	9	x		x	
Barban^37^	2014	16	160 (25%)	Retrieval	19		x	x	
Beck^6^	2014	47	35 (17%)	Retrieval	24		x	x	
Halahalli^57^	2014	18	NA	Maintenance	17	x		x	
Wang^78^	2014	22	32 (20.5%)	Retrieval	10		x	x	
Landsiedel and Gilbert^79^	2014	16	NA	Maintenance	5	x			x
